# Evaluation of leaf rust resistance in the Chinese wheat cultivar ‘Een1’

**DOI:** 10.7717/peerj.8993

**Published:** 2020-05-29

**Authors:** Na Zhang, Lina Zhao, Kahsay Tadesse Mawcha, Chenguang Zhao, Wenxiang Yang, Daqun Liu

**Affiliations:** 1 Technological Innovation Center for Biological Control of Crop Diseases and Insect Pests of Hebei Province, College of Plant Protection, Hebei Agricultrual University, Baoding, Hebei, China; 2 Department of Plant Sciences, Aksum University Shire Campus, Shire, Tigray, Ethiopia; 3 Graduate School, Chinese Academy of Agricultural Sciences, Beijing, China

**Keywords:** Wheat, Leaf rust disease, Leaf rust resistance gene, *Puccinia triticina*, Polymorphism, Molecular mapping, SSR markers, Resistance identification, Inoculation, Gene postulation

## Abstract

Wheat cultivar Een1, 34 near isogenic lines (NILs), and two cultivars were used as plant materials to evaluate the resistance of Een1 to leaf rust disease. Infection type identification and gene postulation were carried out by inoculation of 12 Chinese *Puccinia triticina* (*Pt*) pathotypes. Based on the unique phenotype of Een1, we speculated that Een1 might carry *Lr* gene(s) different from the tested ones. The chromosomal locations for resistance gene to leaf rust disease was employed using SSR primers mapping the populations derived from the cross between Een1 and susceptible Thatcher. A total of 285 plants in the F_2_ population were tested by inoculating *Pt* pathotype FHNQ during the seedling stage. Results from the segregation analysis fits a ratio of 3:1 (${\chi }_{3:1}^{2}=2.37$, *P* = 0.12), indicating the presence of a single dominant gene in Een1 conferring resistance to FHNQ. A total of 1,255 simple sequence repeat (SSR) primers were first used to identify the likely linked markers based on bulk segregation analysis (BSA), and then those likely linked markers were further genotyped in the *F*_2_ population for linkage analysis. Our linkage analysis found that the resistance gene (*LrE1*) was distal to seven SSR loci on the long arm of chromosome 7B, with distances from 2.6 cM (*Xgwm344*) to 27.1 cM (*Xgwm131*). The closest marker *Xgwm344* was further verified with *F*_3_ lines.

## Introduction

Wheat leaf rust, caused by *Puccinia triticina* (*Pt*), is one of the most devastating fungal diseases affecting wheat, causing severe yield losses globally. It has caused serious epidemics in North America and South America, and is a major seasonal disease in India. Destructive epidemics of leaf rust disease occurred in the 1970s in China (Dong, 2001), and in recent years, leaf rust pathogens caused epidemics in major wheat production regions in China including Gansu, Sichuan, Shaanxi, Henan, Anhui, Hebei and Shandong Provinces (Zhou et al., 2013a). These epidemics could be related to global warming and continuous intensive crop production in the same fields. Utilization of resistant cultivars would still be the most effective, economic and eco-friendly way to control wheat leaf rust disease (Chen et al., 1998).

To date, more than 100 leaf rust resistance (*Lr*) genes/alleles have been identified in wheat and its relatives (Singla et al., 2017). Only a few designated genes (*Lr9*, *Lr19*, *Lr24*, *Lr25*, *Lr28*, *Lr29*, *Lr38* and *Lr47*) are effective at the seedlings stage against the prevalent Chinese *Pt* pathotypes (Zhang et al., 2020). The fact that most of these effective genes have not been detected in the Chinese wheat cultivars, means that most cultivars from the Chinese wheat germplasms could be rapidly overcome by these pathogens. It is therefore risky to release cultivars with limited or single gene resistance (also termed as major, seedling or race specific resistance). To cope with the dynamic and rapid evolution of *Pt* populations, it is necessary to identify new and effective resistance genes in different germplasms, so as to enlarge the Chinese wheat gene pool, thereby pyramiding multiple genes in wheat breeding programs.

Due to the advantages of high polymorphism and known chromosome location, simple sequence repeat (SSR) markers have been widely used in genetic studies during the last two decades (Röder et al., 1995). Many *Lr* genes such as *Lr3*, *Lr12*, *Lr37*, *Lr39/41*, *Lr51* and *Lr52* have been mapped on wheat chromosomes using SSR markers (Singh & Bowden, 2011; McIntosh et al., 2013). We have mapped several *Lr* genes in our previous studies including *Lr19* on chromosome 7D (Li et al., 2005), *Lr45* on chromosome 2A (Zhang et al., 2007), *LrZH84*, *LrBi16* and *LrXi* on chromosome 1BL (Zhou et al., 2013b; Zhang et al., 2011; Li et al., 2010a), *LrNJ97* on 2BL (Zhou et al., 2013a), and *LrFun* on chromosome 7BL (Xing et al., 2014). Several adult resistance loci have also been mapped in our previous studies (Qi et al., 2016; Zhang et al., 2017).

Released by Hongmiao State Agricultural Science Research Institute in Hubei province (China), the wheat cultivar Een1 showed high resistance to multiple fungal diseases including leaf rust, stripe rust, stem rust and powdery mildew. This cultivar also has moderate resistance to wheat scab and lodging resistance (https://baike.baidu.com/item/%E9%84%82%E6%81%A91%E5%8F%B7). The objectives of the present study were to (1) investigate the resistance of Een1 to 12 Chinese *P. triticina* pathotypes, and (2) explore and map the leaf rust resistance gene in Een1.

**Table 1:** Seedling infection types on the tested wheat cultivars and NILs when inoculated with 12 *P. triticina* pathotypes.

Cultivars/lines	Pathotypes											
	THST	FHNQ	PHGS	THTQ	THPS	FHTR	SHKN	PHST	THGR	THTS	PHTT	THPS
RL6003 (*Lr1*)	3	;1	3	3	3	;1	4	4	3	3	4	4
RL6016 (*Lr2a*)	3	1	1	3	3	2	4	2	3	3	2	4
RL6047 (*Lr2c*)	3	4	3	3	3	3	4	4	4	3	4	4
RL6002 (*Lr3*)	3	4	3	3	3	3	;	4	4	3	4	4
RL6010 (*Lr9*)	;	;	;	;	;1	;	0	0	;	1	0	0
RL6005 (*Lr16*)	3	4	3	3	3	3	3	3	3	3	4	4
RL6040 (*Lr24*)	;1	;	;1	;	;	;1	;	;1	;1	;	;	;
RL6078 (*Lr26*)	3	3	3	3	3	3	4	4	4	3	4	4
RL6007 (*Lr3ka*)	3	3	;1	3	3	3	;	4	;	3	4	4
RL6053 (*Lr11*)	3	2	3	3	3	3	3	3	4	3	4	2
RL6008 (*Lr17*)	3	3	2	3	3	3	3	3	2	4	4	4
RL6049 (*Lr30*)	1	;1	;1	3	3	3	3	2	;	3	3	3
RL6051 (*LrB*)	3	3	3	3	3	3	4	3	3	3	4	4
RL6004 (*Lr10*)	3	3	3	3	3	3	;	4	4	3	4	4
RL6013 (*Lr14a*)	3	2	4	1	3	2	3	3	2	4	4	4
RL6009 (*Lr18*)	3	2	1	1	2	3	;	3	4	3	3	2
RL6019 (*Lr2b*)	2	3	1	3	3	3	4	3	4	3	4	3
RL6042 (*Lr3bg*)	3	4	3	3	3	3	;	3	4	3	4	3
RL6006 (*Lr14b*)	3	3	4	3	3	3	4	4	4	4	4	4
RL6039 (*Lr14ab*)	3	;1	1	1	3	2	3	3	2	3	4	4
RL6052 (*Lr15*)	3	;1	3	3	3	3	;	4	3	3	;	4
RL6040 (*Lr19*)	;	;	;	0	;	;	;	;	;	1	0	;
RL6043 (*Lr21*)	3	2	3	3	2	2	4	3	;	3	4	4
RL6012 (*Lr23*)	3	3	4	3	3	3	4	3	4	4	4	4
RL6084 (*Lr25*)	3	3	3	3	3	3	4	4	4	4	4	4
RL6079 (*Lr28*)	;	;	;	;	;	;	;	;1	;1	;	;	;
RL6080 (*Lr29*)	3	;	3	3	3	3	;	;	;	;	;	;
RL6057 (*Lr33*)	3	3	3	3	3	3	3	3	4	3	4	4
E84018 (*Lr36*)	0	;	3	1	;	;1	4	1	3	3	4	4
RL6097 (*Lr38*)	;1	;	;1	;	;	;	;	;1	0	;	;	;
KS86WGRC02 (*Lr39*)	3	;	3	3	3	1	2	2	2	;	;	;
KS91WGRC11 (*Lr42*)	;1	3	;	;	;	;	;	;	2	;	3	;
RL7147 (*Lr44*)	4	3	2	2	3	3	3	3	3	2	4	4
KS96WGRC36 (*Lr50*)	4	1	3	3	3	3	3	3	4	4	3	4
Een1	;	;	;	;	;	;	;	1	2	3	3	4
Bimai 16	1	1	1	;1	1	2	;	3	;1	0;	3	;
Fundulea 900	0;	;1	;	0;	;	;1	1	;1	0;	4	0;	0;
Thatcher	4	4	4	4	4	4	4	4	4	4	4	4

## Materials & Methods

### Plant materials and leaf rust evaluation in the greenhouse

Experimental plant materials including 34 near-isogenic lines (NILs) with Thatcher background, susceptible line Thatcher, cultivar Een1, Bimai16 and Fundulea900 were used for gene allelic polymorphism evaluations (Table 1). Five to seven seedling plants for each tested line were grown in a growth chamber and the inoculations were performed by spraying urediniospores of the tested 12 *Pt* pathotypes when the first leaves appeared fully expanded. Inoculated seedlings were placed in a chamber and incubated at 20 °C with 100% relative humidity in dark for 16 h. They were then transferred to a greenhouse with 12 h light/12 h darkness at 22 ± 3 °C with 70% relative humidity (RH). Infection types (ITs) were scored at 14 days post inoculation according to the Stakman scale modified by Roelfs, Singh & Saari (1992). The standards applied in this scale were: “0” representing immunity (no sign of infection), “;” for necrotic flecks, “1” for small uredinia surrounded by a necrosis, “2” for small to medium uredinia surrounded by chlorosis, “3” for medium uredinia without chlorosis or necrosis, and “4” for large uredinia without chlorosis or necrosis. Plants that scored IT 3 or higher were considered susceptible. The F_1_ population (15 plants), F_2_ population (285 plants) and F_3_ population (30 seedlings from each of the 155 plants selected from the F_2_ population), derived from Een 1 ×Thatcher, were used for mapping to identify SSR molecular markers associated with the leaf rust resistance genes. *Pt* pathotype FHNQ was used to phenotype all the plants tested in the F_1_, F_2_ and F_3_ populations.

### Simple Sequence Repeat (SSR) marker analysis

Genomic DNAs were extracted from seedlings using the CTAB method. A total of 1255 SSR primers (Zhang et al., 2011) were selected randomly from the GWM, WMC, CFA, CFD, and BARC primer series covering each wheat chromosome (https://wheat.pw.usda.gov/). All the primers were synthesized by Sangon Biotech (Shanghai) Co., Ltd. Bulked segregant analysis described by Michelmore, Paran & Kesseli (1991) (equal amounts of genomic DNA from 10 resistant (Br) and 10 susceptible (Bs) from F_2_ plants, along with the two parents) was performed in a preliminary screen to identify molecular markers likely to be linked to the resistance gene in Een1. Polymorphic primers for the parents and the bulked pools were then genotyped across the individual lines in the F_2_ and F_3_ populations. PCR amplification was carried out as described by Xing et al. (2014). Mixed with 8 µL formamide loading buffer, all the denatured PCR products were separated on 6% denaturing polyacrylamide gels for approximately 1.5 h at 100 W and viewed by the silver staining method.

### Construction of the linkage map

To evaluate the deviations of the observed and expected segregation ratios, Chi-squared (*χ*^2^) tests for goodness-of-fit were calculated using Microsoft Excel 2010 software. Linkage analysis between SSR markers genotyping and the phenotyping results were performed by MapManager QTXb20 software (LOD = 3) (Manly, Cudmore & Meer, 2001). And Kosambi mapping function (Kosambi, 1944) was used to calculate the genetic distances between the markers and the resistance gene. The linkage map was drawn using Mapdraw Version 2.1 software (Liu & Meng, 2003).

## Results

### The gene postulation for Een1

Twelve Chinese *Pt* pathotypes (THST, FHNQ, PHGS, THTQ, THPS, FHTR, SHKN, PHST, THGR, THTS, PHTT, THPS) were used to phenotype the wheat cultivar Een1, susceptible line Thatcher and 34 near isogenic lines (NILs) carrying different *Lr* genes. Two other wheat cultivars, Fundulea900 and Bimai16, were also tested. Results for all the phenotypic infection types (IT) are listed in Table 1. Een1 showed high resistance to nine of the tested *Pt* races (THST, FHNQ, PHGS, THTQ, THPS, FHTR, SHKN, PHST, THGR) whilst three *Pt* races (THTS, PHTT and THPS) showing virulence. An overall analysis showed that *Pt* race THTS had low IT records for the near-isogenic lines including TcLr9, TcLr24, TcLr19, TcLr28, TcLr29, TcLr38, TcLr39, TcLr42 and TcLr44. The *Pt* race PHTT produced low ITs with TcLr2a, TcLr9, TcLr24, TcLr15, TcLr19, TcLr28, TcLr29, TcLr38, and TcLr39 lines. Pathotype THPS recorded a low infection type on TcLr9, TcLr24, TcLr11, TcLr18, TcLr19, TcLr28, TcLr29, TcLr38, TcLr39, and TcLr42. The combined results from these three *Pt* races enable us to conclude that none of the corresponding genes including *Lr2a*, *Lr9*, *Lr11*, *Lr15*, *Lr18*, *Lr19*, *Lr24*, *Lr28*, *Lr29*, *Lr38*, *Lr39*, *Lr42* and *Lr44* existed in our tested wheat cultivar Een1.

With a different infection type (“;”) on TcLr3ka, TcLr30 and TcLr21 lines, the *Pt* race THGR showed infection type “2” on Een1, we can conclude that cultivar Een1 carried no *Lr3ka*, *Lr30* and *Lr21* genes. The general results in Table 1 reveal that the phenotype of Een1, in relation to the pathotypes, had different result patterns for TcLr1, TcLr2c, TcLr2b, TcLr3, TcLr3bg, TcLr10, TcLr17, TcLr14ab, TcLr14a, TcLr36 and TcLr50 lines. These results indicate that Een1 carried none of the genes mentioned above. In this study, final conclusions for genes in Een1 with high IT records on corresponding NILs, *Lr2c*, *LrB*, *Lr16*, *Lr26*, *Lr14b*, *Lr23*, *Lr25* and *Lr33* were not possible. Based on the combined phenotyping results, it could be concluded that Een1 may carry new *Lr* gene(s) besides one or several of *Lr2c*, *LrB*, *Lr16*, *Lr26*, *Lr14b*, *Lr23*, *Lr25* or *Lr33*. In addition, depending on the unique phenotypes of Een1 responding to the 12 tested *Pt* pathotypes, we questioned whether wheat cultivar Een1 may carry unknown leaf rust resistance gene(s) other than the tested *Lr* genes, or there may be a combined action of more than one *Lr* gene.

### Inheritance of leaf rust resistance in Een1

All the plants in the F_2_ and *F*_3_ populations, along with the parents, were inoculated with *Pt* pathotype FHNQ (avirulent on Een1 and virulent on Thatcher) at the seedling stage and the test results were presented in Table 2 and Table 3. Of all the 285 plants tested in F_2_ population, 225 individuals showed resistance phenotype and 60 were susceptible, giving a suitable 3:1 ratio (*χ*^2^_3:1_ = 2.37, *P* = 0.12) (Table 2). Among the tested 155 families in the F_3_ population, 40 were homozygous resistant, 80 heterozygous and 35 homozygous susceptible, fitting an expected ratio of 1:2:1 (*χ*^2^_1:2:1_ = 0.48, *P* = 0.78) (Table 3). Results from both the F_2_ and F_3_ populations indicated that leaf rust resistance to *Pt* FHNQ in wheat cultivar Een1 was conferred by a single dominant gene, tentatively designated as *LrE1.*

**Table 2:** Segregation of seedling reactions to *Pt* race FHNQ in Een1, Thatcher and their F_**1**_ and F_**2**_ progenies.

Material	Total plant	Resistance	Susceptible	Goodness of fit test
Een1	15	15		
Thatcher	15		15	
F_1_	15	15		
F_2_	285	225	60	*χ*^2^_3:1_ = 2.37, *P* = 0.12

**Table 3:** Phenotypes and genotypes inferred from reactions of F_**3**_ lines inoculated with Pt race FHNQ and the corresponding alleles at SSR loci Xgwm344.

F_**3**_ phenotype	F_**3**_ genotype	Goodness of fit test	Allele	
			D	B
Resistance 120	RR 40		39	1
	Rr 80	*χ*^2^_1:2:1_ = 0.48, *P* = 0.78	78	2
Susceptible 35	rr 35		4	31

**Notes.**

RR homozygous resistant Rr segregating rr homozygous susceptible D homozygous for Een1 allele or heterozygous B homozygous for Thatcher allele

### Linkage analysis and genetic map

**Figure 1:**
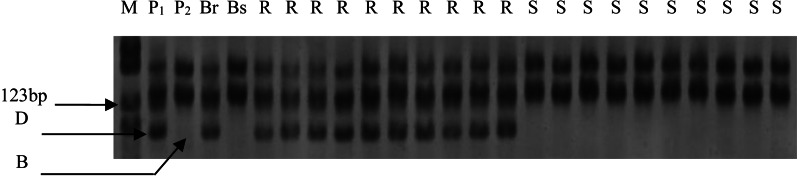
Electrophoresis of PCR products amplified with X*gwm344* on polyacrylamide gels. M: PBR322/*Msp* I Marker; P_1_: resistance parental line Een1; P_2_: susceptible parental line Thatcher; Br: resistance bulk; Bs: susceptible bulk; R: resistance F_2_ plants; S: susceptible F_2_ plants; B: homozygous for Thatcher allele; D: homozygous for Een1 allele or heterozygous.

**Figure 2:**
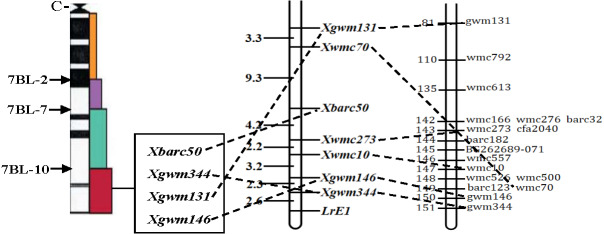
Linkage map of the resistance gene* LrE1* using SSR markers on chromosome 7BL. Deletion bin map on wheat chromosome 7BL (Sourdille et al., 2004) (left); linkage map of leaf rust resistance gene *LrE1* generated using data from F_2_ population of Een1 × Thatcher (centre). Locus names and corresponding locations are indicated on the right, genetic distances are labeled on the left in centiMorgans; and compared with previously published wheat chromosome 7BL map (Somers, Isaac & Edwards, 2004) (right).

Out of the 1255 randomly selected wheat SSR primers, seven SSR primers (*Xgwm344*, *Xgwm146*, *Xwmc10*, *Xwmc70*, *Xwmc273*, *Xbarc50*, *Xgwm131*) located on chromosome 7BL showed polymorphism between the resistance and the susceptible bulks as well as the parents (Table 4). All the seven polymorphic SSR primers were further used to genotype the DNA samples from each of the 285 F_2_ plants (Fig. 1). The linkage analysis using Mapmanager QTXb20 software showed that *Xgwm344* was linked to *LrE1* with a genetic distance of 2.6 cM as the closest SSR marker (Fig. 2). The SSR marker *Xgwm146* had a value of 4.9 cM, thus scoring the second genetic distance. And the furthest marker was *Xgwm131* with 27.1 cM from the gene *LrE1*. In the 155 families in the F_3_ population, *Xgwm344* proved to be 4.8 cM to gene *LrE1*.

**Table 4:** F_**2**_ phenotypes and the corresponding alleles at SSR markers loci.

Marker loci	F_2_ phenotype	Allele				
		A	H	B	D	B
*Xgwm344*	Resistance	–	–	–	222	3
	Susceptible	–	–	–	4	56
*Xgwm146*	Resistance	76	143	6	–	–
	Susceptible	1	6	53	–	–
*Xwmc10*	Resistance	70	138	17	–	–
	Susceptible	1	7	52	–	–
*Xwmc273*	Resistance	–	–	–	208	17
	Susceptible	–	–	–	14	46
*Xbarc50*	Resistance	53	154	18	–	–
	Susceptible	1	24	35	–	–
*Xwmc70*	Resistance	78	126	21	–	–
	Susceptible	1	46	13	–	–
*Xgwm131*	Resistance	–	–	–	197	28
	Susceptible	–	–	–	46	14

**Notes.**

D homozygous for Een1 allele or heterozygous B homozygous for Thatcher allele A homozygous for Een1 allele H heterozygous

## Discussion

Seedlings of the winter wheat cultivar Een1 showed high resistance to multiple fungal diseases. With good agronomic traits, this cultivar has significant potential for the future genetic improvement in wheat. Results from this study indicated that Een1 has a high resistance to several epidemic *Pt* pathotypes including THST, FHNQ, PHGS, THTQ, THTS, FHTR and SHKN. Specific genetic analysis on the F_2_ and F_3_ populations indicated that the resistance of Een1 against FHNQ was conferred by a single dominant gene, provisionally designated as *LrE1*. This resistance locus was distal to the seven SSR markers (*Xgwm344*, *Xgwm146*, *Xwmc10, Xwmc273*, *barc50*, *Xwmc70*, *Xgwm131*) on the long arm of chromosome 7B. The closest SSR marker, *Xgwm344*, was linked to *LrE1* with a genetic distance of 2.6 cM. The linear order for these markers in the genetic map drawn in this study was similar to the high-density consensus map developed by Somers, Isaac & Edwards (2004), with the exception that *Xwmc70* was proximal to *Xgwm344* and *Xgwm146*. The difference between these results could be due to the specific populations analyzed or the molecular markers employed in the tests.

Currently, there are five designated *Lr* genes on wheat chromosome 7B (*Lr14a*, *Lr14b*, *Lr68*, *LrBi16*, and *LrFun*) (Herrera-Foessel et al., 2008; Dyck & Samborski, 1970; Herrera-Foessel et al., 2012; Zhang et al., 2011; Xing et al., 2014). *LrE1* is a resistance gene activated at the seedling stage, since *Lr68* has been previously reported as an adult resistance gene (Herrera-Foessel et al., 2012), this indicates that *LrE1* is different from *Lr68*. Since wheat near isogenic lines (NILs) carrying *Lr14a* or *Lr14b* showed different phenotypes than Een1 when inoculated with the FHNQ, *LrE1* could be different from *Lr14a* and *Lr14b*. Een1 had similar resistant reaction to FHNQ as Bimai16 and Fundulea900. However, Zhang et al. (2011) reported that the *LrBi16* in Bimai16 was flanked between molecular markers *Xcfa2257* (2.8 cM) and *Xgwm344* (2.9 cM), with *Xgwm146* at the same side of the chromosome. The *LrFun* gene in wheat cultivar Fundulea900 was located between molecular markers *Xgwm344* (4.4 cM) and *Xwmc70* (5.7 cM) (Xing et al., 2014). It seems like the *LrE1* gene has a different chromosome position, which is outside the region of *Xgwm344* and *Xwmc70*. Therefore, *LrE1* might be different from both *LiBi16* and *LrFun*. Future studies are needed to further clarify the genetic relationship between these three genes*,* which would include phenotyping and fine mapping on the populations derived from the crosses between Een1 and the lines with single *Lr* gene including *LrBi16* and *LrFun* as what has been done by Zhang et al. (2015). Since only the *Pt* race FHNQ was tested in this study, other low virulence *Pt* pathotypes such as THST, PHGS, THTQ should also be the focus in the future.

Based on previous research findings, only a few *Lr* genes, including *Lr1*, *Lr3*, *Lr3bg*, *Lr10*, *Lr13*, *Lr14a*, *Lr16*, *Lr23*, *Lr26*, *Lr34* and *Lr35*, were detected in Chinese cultivars. Most of the *Lr* genes are likely to lose their resistance function due to the rapid evolution of *Pt* pathotypes in China (Li et al., 2010b). The pedigree of Een1 are Lvorin10/761//Sumai3. No *Lr* gene has been found in 761. Sumai3 contained *Lr1* and *Lr34*, and showed slow-rusting resistance at the adult stage with a disease index (DI) of 0.8 in the field (Ding et al., 2010), Een1 showed slow-rusting resistance to mixed *Pt* pathotypes at the adult stage (Fig. S2), so Een1 may have inherited *Lr34* (on chromosome 7D) from Sumai3. Previous research showed that Een1 has a 1BL.1RS and carries another leaf rust resistance gene *Lr26* on chromosome 1B (Li et al., 2010b; Yan et al., 2017). According to Hu & Chen (1992), there were/was more *Lr* gene(s) in Lovrin10 besides *Lr26* and *Lr2c*, so the resistance to FHNQ in Een1 may be derived from Lovrin10. Generally, utilization of wheat cultivars carrying multiple resistance genes is an effective way to improve both wide-spectrum resistance against various pathogens and durability of such resistance. The cultivar Een1, with its characteristic of multi-resistance (with the known genes *Lr26* and *LrE1* identified in this paper) and other good agronomic traits, could be widely distributed in China to delay the “loss of resistance”.

## Conclusions

A seedling leaf rust resistance gene (provisionally named *LrE1*) was identified in Een1, which showed high resistance to nine *Puccinia triticina* (*Pt*) pathotypes prevalent in China. With multi-resistance traits and slow-rusting resistance, the cultivar could become important in delaying loss of disease resistance if widely distributed.

##  Supplemental Information

10.7717/peerj.8993/supp-1 Figure S1 Phenotypes for some of the tested materials

10.7717/peerj.8993/supp-2 Figure S2 Phenotypes of Een1 at adult stage

## Additional Information and Declarations

### Data Availability

The following information was supplied regarding data availability: All the data are available in the Tables.
