# Supplementary figures and images for: Evaluation of leaf rust resistance in the Chinese wheat cultivar ‘Een1’
Source: PeerJ. 2020 May 29;8:e8993. doi: 10.7717/peerj.8993 (PMC7263293; doi:10.7717/peerj.8993)

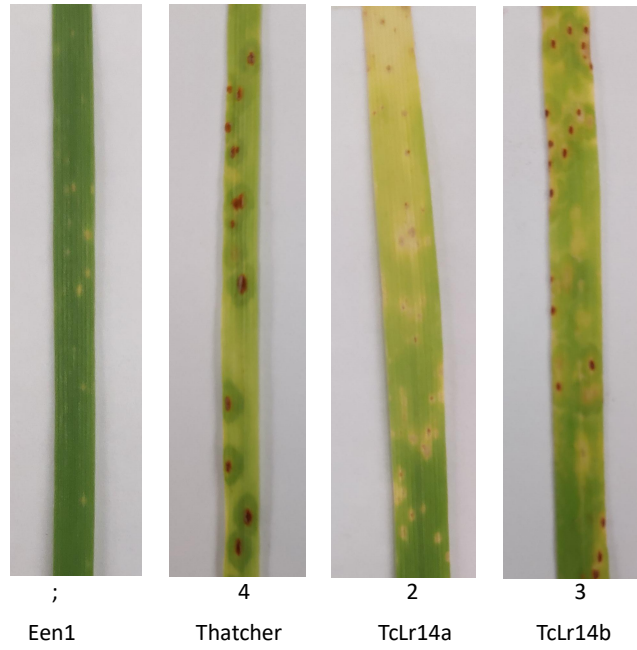

**Fig. 1** Phenotypes for some of the tested materials

Supplement: Figure S1 [file peerj-08-8993-s001.pdf]

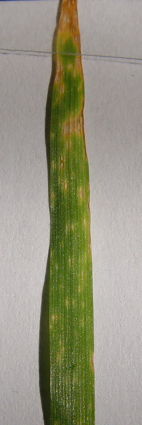

Supplement: Figure S2 [file peerj-08-8993-s002.png]
